# One Pulmonary Lesion, 2 Synchronous Malignancies

**DOI:** 10.1177/2324709618785934

**Published:** 2018-06-28

**Authors:** Masooma Aqeel, Nevin Uysal-Biggs, Timothy S. Fenske, Nagarjun Rao

**Affiliations:** 1 Aga Khan University, Karachi, Pakistan; 2 Medical College of Wisconsin, Milwaukee, WI, USA; 3 Aurora St. Luke’s Hospital, Milwaukee, WI, USA

**Keywords:** cavitary nodule, lung cancer, mantle cell lymphoma, collision histology, non-Hodgkin’s lymphoma

## Abstract

*Introduction*. Mantle cell lymphoma (MCL) comprises approximately 3% to 10% of all non-Hodgkin lymphomas. Although there is an increased risk for secondary malignancies after treatment among non-Hodgkin lymphomas survivors, a synchronous diagnosis of primary lung cancer arising in conjunction with lymphoma at the same site has rarely been reported. We report an unusual case of primary lung adenocarcinoma with coexistent MCL within the same lung lesion. *Case Presentation*. A 55-year-old female with newly diagnosed stage IV-B MCL was referred for workup of a right upper lobe cavitary lesion detected during lymphoma staging. A whole-body positron-emission tomography-computed tomography scan revealed diffuse adenopathy but also identified a cavitary right upper lobe lesion atypical for lymphoma. Bronchoscopy was unremarkable with cytology (on lavage) negative for malignancy. At 2 months, a computed tomography scan of the chest showed a persistent lesion. A video-assisted thoracoscopic wedge resection was performed. Histopathological examination revealed a lepidic predominant, well-differentiated adenocarcinoma (stage T1a) and foci of lymphoid infiltrate within and adjacent to the adenocarcinoma consistent with lung involvement by MCL. *Discussion*. Synchronous presentation of primary lung adenocarcinoma and lymphoma at a single site is exceedingly rare. Nonresolving pulmonary lesions with features atypical for lymphoma should be viewed with caution and worked up comprehensively to rule out occult second malignancies, in order to guide a prompt diagnosis and appropriate treatment.

## Introduction

According to the World Health Organization classification of 2008,^1^ mantle cell lymphoma (MCL) is classified as a mature B-cell lymphoma, comprising about 6% of all adult non-Hodgkin’s lymphoma (NHL) in the United States. MCL is aggressive and usually diagnosed at an advanced stage of the disease in middle-aged to older patients. Its incidence has risen over recent years and is noted to be highest among males, Caucasians, and older patients (median age 67 years at diagnosis).^2^

Although an increased long-term risk for developing secondary neoplasms, including lung cancer, has also been described among NHL survivors,^3,4^ a histopathological diagnosis of 2 synchronous malignancies at the same site is exceedingly rare. In this article, we present the case of a middle-aged female diagnosed with MCL and primary pulmonary adenocarcinoma within the same nodule.

## Case Report

**Figure 1.**
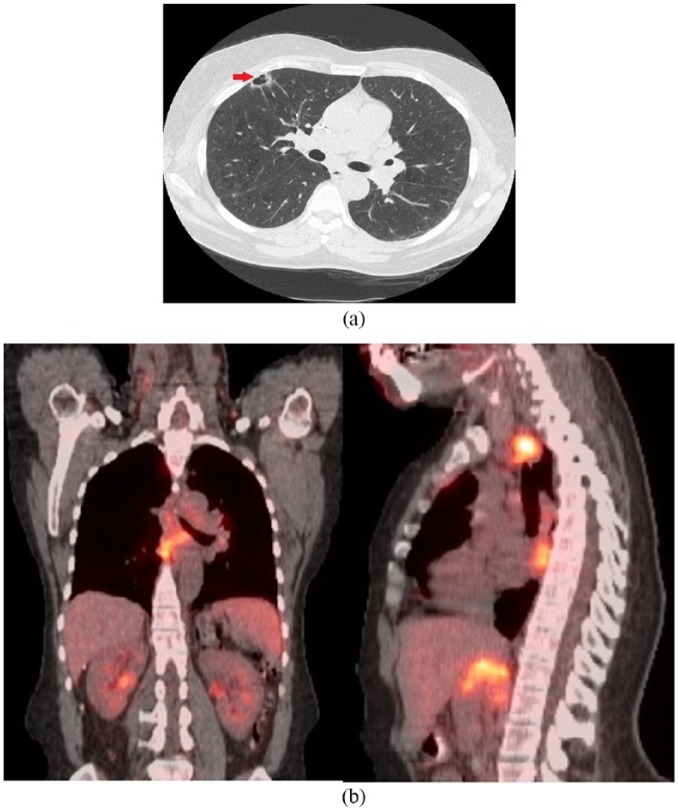
(a) Computed tomography scan of chest showing a 1.7 × 1.1 cm right upper lobe cavitary nodule. (b) Positron-emission tomography scan showing extensive lymphadenopathy both above and below the diaphragm but no uptake in the right upper lobe cavitary lesion.

A 55-year-old female presented to the emergency department for evaluation of severe lower flank pain radiating to her lower abdomen and chest. Further review of symptoms revealed that she also had cough, night sweats, chills, and an unintentional weight loss of 31 pounds over 3 months. Clinical examination was significant for bilateral axillary lymphadenopathy. Subsequently, a computed tomography (CT) scan of her chest, abdomen, and pelvis was performed that revealed extensive bilateral lymphadenopathy (above and below the diaphragm) as well as a new right upper lobe (RUL) thin-walled cavitary lung lesion with spiculated margins (Figure 1a). A positron-emission tomography (PET)-CT scan showed highly metabolically active lymphadenopathy in the neck, chest, abdomen, and pelvis but minimal to no PET avidity within the RUL cavitary lesion (Figure 1b). Axillary lymph node sampling showed moderate-to-large B-lymphocytes (positive for CD5, CD20, and cyclin D1), with fluorescence in situ hybridization positive for t(11; 14), consistent with MCL. Although a bone marrow biopsy revealed low disease burden (<10% involvement), her lymphoma demonstrated a high proliferation rate (Ki67 proliferation index 30%), and she was diagnosed with stage IV-B MCL.

**Figure 2.**
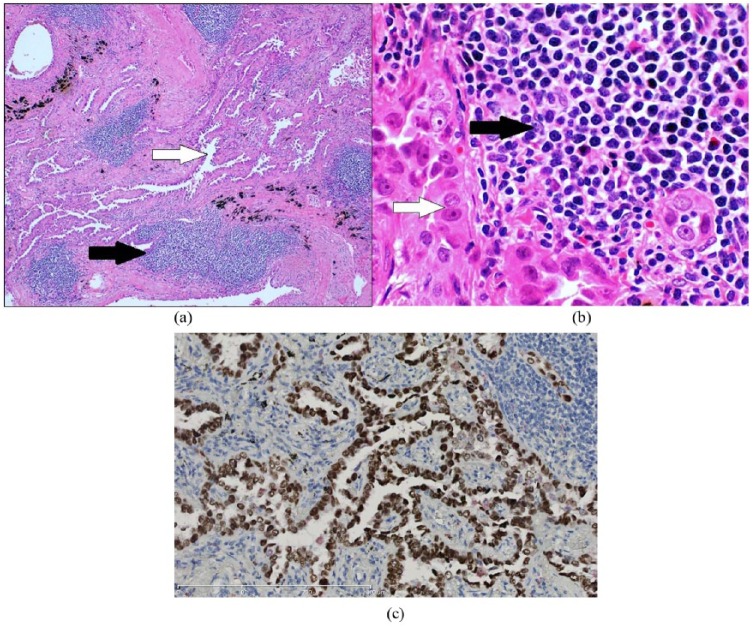
(a) Histopathological examination revealed an infiltrating lepidic predominant adenocarcinoma (white arrow) with coexistent foci of lymphoid infiltrate (black arrow). (b) High-power examination revealed small, irregular, convoluted lymphocytes comprising the lymphoid infiltrate (black arrow). Adenocarcinoma cells (white arrow) show atypical, large nuclei with prominent nucleoli (hematoxylin and eosin). (c) Immunohistochemistry showing strong positivity for thyroid transcription factor-1.

**Figure 3.**
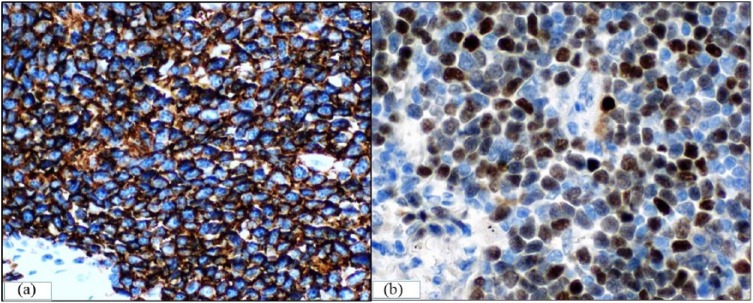
Immunohistochemistry revealed lymphocytes showing strong and diffuse positivity for (a) generic B-cell marker CD20 and (b) mantle cell lymphoma (MCL) marker cyclin D1.

Prior to initiation of chemotherapy, she was referred to the pulmonary clinic for workup of the lung lesion, which was felt to have radiographic features atypical for lymphoma. She was an active, 30 pack-year smoker with symptoms of stable chronic bronchitis, sinusitis, and scant hemoptysis. Serum tuberculosis testing (TB-quantiferon) was negative. Although no prior self-history of cancer, she had a strong family history for cancer (lung cancer [father, paternal uncle, and paternal grandfather], cervical cancer [mother], and premenopausal breast cancer [paternal aunt]). Chest CT was significant for severe emphysema, multiple indeterminate pulmonary nodules, and a 1.7 × 1.1 cm, subpleural, thin-walled cavitary lesion with spiculated margins in the RUL (Figure 1a). Initial differential diagnosis included malignancy (primary/metastatic carcinoma and less likely lymphoma), subacute or chronic infections (such as tuberculosis and fungal infection), and vasculitis. Bronchoscopy was unremarkable, and bronchoalveolar lavage showed 87% macrophages, 10% neutrophils, and 2% lymphocytes, without malignant cells. Respiratory cultures grew 1+ *Aspergillus flavus*. Serum antineutrophil cytoplasmic antibodies and rheumatoid arthritis factor were negative. A short-interval (2 months) follow-up CT imaging showed stable pulmonary nodules but a persistent RUL cavitary lesion. A video-assisted thoracoscopic wedge resection was performed to exclude other diagnoses prior to initiation of chemotherapy for MCL. Pathological examination revealed a lepidic predominant, well-differentiated adenocarcinoma (pathological stage T1a), with coexistent foci of lymphoid infiltrates within and adjacent to adenocarcinoma (Figure 2a). The lymphoid infiltrate had histological features (convoluted irregular small nuclei; Figure 2b). By immunohistochemistry, the adenocarcinoma component was strongly positive with thyroid transcription factor-1, confirming pulmonary origin (Figure 2c) and discounting the possibility of metastasis from a possible mammary carcinoma. In addition, immunohistochemical profile (positive for B-cell markers CD20, pax5, and BCL1 [cyclin D1]; Figure 3a and b) was consistent with MCL. Multidisciplinary consensus was that no additional surgery, lymph node sampling, or adjuvant chemotherapy was needed. She was subsequently observed for both adenocarcinoma and lymphoma (high proliferation rate but with low disease burden), with surveillance imaging. A year later, however, she presented with dyspnea and a new right-sided pleural effusion (cytology positive for adenocarcinoma, negative for epithelial growth factor receptor/anaplastic lymphoma kinase). She received 6 cycles of carboplatin/pemetrexed/bevacizumab with good treatment response; however, she declined further maintenance therapy due to an intolerance of side effects. A few months later (a year ago), she presented with weight loss, chills, and night sweats, and CT imaging showed progressive lymphadenopathy concerning for MCL. She received ibrutinib and rituximab with good response initially; however, she could not complete the prescribed therapy due to side effects and was unfortunately lost to follow-up at the time of this writing.

## Discussion

Our case highlights 2 important findings. First, this is a very rare case of synchronous primary lung cancer and MCL found within the same cavitary lung nodule. Second, this case highlights important features of lung lesions/nodules that would be considered “unusual or atypical” for lymphomatous involvement of the lung and raise concern for a possible secondary process in the lung.

Large population-based cancer registry reviews show that NHL survivors are at a significantly greater risk for secondary malignancies than the normal population (all solid tumors; observed to expected ratio = 1.37).^3^ This risk remains elevated even up to 30 years after treatment for NHL and is independent of the use of external beam radiation therapy.^4^ Lymphomas and leukemias tend to be among the most common neoplasms involved even as subsequent neoplasms, and usually arise within the first 5 years.^5^ Solid tumors are more common after 5 years, with breast and lung cancer being the most frequent.^6^ In a review of 77 823 patients, Tward et al demonstrated that NHL survivors have an absolute excess cancer risk of 6.36 times (in men) and 7.10 times (in women) for a second primary lung and/or mediastinal tumor, specifically.^4^

Despite these increased risks, however, a diagnosis of a synchronous second malignancy with a coexistent lymphoproliferative disorder is still quite rare. Synchronous multiple primary cancers are defined as 2 or more primary tumors occurring within 6 months of each other.^7,8^ In reviewing the medical literature, it appears that an association between NHL and breast cancer is well recognized. Wiernik et al, in a large series of 87 cases, established that in women with both breast cancer and NHL, lymphoma is significantly more likely to be diagnosed after or concurrently with breast cancer. The 2 malignancies could be seen in separate organs, or as “collision tumors,” with coexistence in anatomic proximity to each other.^9^ Experimental evidence also appears to suggest that breast cancer and lymphoma of the breast may share some common biologic characteristics.^10^ However, we found only a handful of case reports that document synchronous NHL and primary lung cancer.^11-13^ Within NHL, MCL appears to be more commonly associated with secondary genitourinary malignancies rather than lung cancer.^14^ Where authors report synchronous MCL and primary lung cancer, it is either at separate sites^15^ or in the form of metastases to the same regional lymph node.^16^ We found only 2 reports describing synchronous involvement of same thoracic site (ie, pleura) by both MCL and primary lung cancer^17,18^ (summarized in Table 1). Our case adds itself to a rare but growing list of reports of synchronous MCL and primary lung cancer, and is also the first, to our knowledge, to report synchronous MCL and primary lung cancer within the same cavitary lung nodule (“collision histology”).

**Table 1.** Case Reports Citing Synchronous Occurrence of Non-Hodgkin’s Lymphoma and Primary Lung Cancer.

Title	Authors	Year	Site	Case Description
Coexistence of non-Hodgkin’s lymphoma and non–small-cell lung carcinoma: diagnosis and treatment	Rothenburger et al^11^	2001	Different sites; lung mass and lymph nodes	A 67-year-old NHL survivor developed new diffuse lymphadenopathy and a pulmonary mass. Inguinal lymph node biopsy revealed relapsed NHL. Due to lack of treatment response seen in the pulmonary mass, a complex lobectomy was performed and revealed a secondary non–small-cell lung cancer.
Synchronous pulmonary adenocarcinoma and extranodal marginal zone/low-grade B-cell lymphoma of the MALT type	Chanel et al^12^	2001	Same site (pulmonary mass)	An interesting case of a 74-year-old male with a nonresolving pulmonary mass that was surgically removed to reveal synchronous adenocarcinoma and extranodal low-grade B-cell lymphoma of the MALT type.
Mantle cell lymphoma in lymph nodes with metastatic small cell carcinoma of lung: a diagnostic and treatment dilemma	Kampalath et al^16^	2004	Same site (lymph nodes)	A 58-year-old female with metastatic small cell lung cancer with synchronous mantle cell lymphoma.
A rare tumoral combination, synchronous lung adenocarcinoma and mantle cell lymphoma of the pleura	Hatzibougias et al^18^	2008	Same site (pleura)	A 73-year-old male who underwent a bullectomy for persistent pneumothorax was incidentally diagnosed with synchronous pulmonary adenocarcinoma and MCL of the pleura.
Synchronous pulmonary squamous cell carcinoma and mantle cell lymphoma of the lymph node	Sun et al^15^	2011	Different sites; lung mass and lymph nodes	A 57-year-old male presenting with a right lower lobe pulmonary mass and cervical and mediastinal lymphadenopathy. The lung mass was biopsied to show squamous cell lung cancer and the lymphadenopathy revealed MCL.
Synchronous BALT lymphoma and squamous cell carcinoma of the lung: coincidence or linkage?	Oikonomou et al^13^	2013	Different sites; 2 nodules in separate lobes	A 72-year-old male presents with 2 large masses (one in the right middle lobe and another in left lower lobe). Biopsy of the left lobe revealed a BALT lymphoma. When the right mass did not respond to lymphoma therapy, a second biopsy was done and revealed concomitant squamous cell lung cancer.
Synchronous pulmonary malignancies: atypical presentation of mantle cell lymphoma masking a lung malignancy	Masha et al^17^	2015	Same site (pleura)	A 70-year-old male presented with a large right pleural effusion. Pleural fluid analysis clonal populations consistent with mantle cell lymphoma. He underwent treatment for lymphoma with no improvement. A CT-guided biopsy then revealed tissue consistent with undifferentiated carcinoma, favoring lung primary.

Abbreviations: NHL, non-Hodgkin’s lymphoma; MALT, mucosa-associated lymphoid tissue; MCL, mantle cell lymphoma; BALT, bronchus-associated lymphoid tissue; CT, computed tomography.

The biological mechanisms behind the pathogenesis of such synchronous multiple primary malignancies are still largely unknown. Authors have proposed certain mechanisms such as the possible spread of cancerous cells from a single clone to multiple sites,^19^ genetic defects involving mismatch repair systems,^20^ a positive family history of malignancy,^8^ and/or a complex interplay of genetics, impaired immunity, viremia (ie, Epstein-Barr virus, etc), and carcinogenic risk factors (such as smoking) that ultimately all contribute to the development of synchronous multiple primary cancers.^5^ It is plausible that both complex genetics and history of smoking placed our patient at risk for these 2 tumors.

Second, this case highlights some important diagnostic and radiological considerations. It is conceivable that the detection of a second pulmonary malignancy was simply an incidental finding in our patient (a smoker) and that a lymphoma staging workup essentially replaced what could have been routine age-appropriate lung cancer screening otherwise (our center sees about 2 to 3 such cases annually [personal communication]). However, this case emphasizes that it is also important to be cognizant of the “usual” or “typical” patterns of lung involvement with lymphoma. Extranodal thoracic involvement is not uncommon with NHL and may represent either primary pulmonary lymphoma or secondary spread. Common radiographic patterns of lymphomatous pulmonary involvement include diffuse interstitial thickening, mass-like consolidation with homogenous attenuation, air-bronchograms and preserved vascular markings, perilymphatic nodularity, and PET avidity in the lung similar to other known sites involved with NHL.^21^ This lesion was atypical given its isolated, peripheral, “cavitary” nature and without any significant PET avidity in comparison with surrounding lymphadenopathy (Figure 1b). Instead, an extensive smoking history coupled with radiographic findings of a single cavitary lesion with spiculated margins, measuring more than 10 mm in size, made her odds of having a potential secondary process such as a lung malignancy much more likely.

It is also important to note an “unresolving” nature of certain pulmonary lesions/nodules. In several prior published reports, we observe that an initial diagnosis is often reached via a biopsy of a certain site. However, it is the “unresolving” nature or “lack of a complete response” to appropriate therapy that often prompts physicians to search for an alternate or synchronous diagnosis.^11-13,17^

In summary, we describe here a very unusual case of MCL and primary pulmonary adenocarcinoma presenting synchronously within one cavitary lung lesion. Synchronous malignancies such as these are exceedingly rare and crucial to identify promptly as treatment can be markedly different. This case highlights the importance of a high index of clinical suspicion of lesions that do not resolve on short-term follow-up (either spontaneously or with treatment) and a keen radiologic awareness of the features that would be unusual for lymphomatous involvement of the lung.
